# A Systematic Review of the Acute Effects of Exercise on Immune and Inflammatory Indices in Untrained Adults

**DOI:** 10.1186/s40798-015-0032-x

**Published:** 2015-10-20

**Authors:** William M. C. Brown, Gareth W. Davison, Conor M. McClean, Marie H. Murphy

**Affiliations:** Sport and Exercise Sciences Research Institute, Ulster University, Jordanstown, BT37 0QB Northern Ireland

## Abstract

**Background:**

Cardiovascular disease (CVD) is the leading cause of global mortality. Although the incidence may be reduced with regular exercise, the health benefits of a single bout of exercise on selected CVD risk factors are not well understood. The primary objective of this review is to consider the transient effects of exercise on immune (neutrophil count) and inflammatory (interleukin-6 [IL-6], C-reactive protein [CRP]) markers in untrained adults.

**Methods:**

MEDLINE, EMBASE, CINAHL, Sports Discus and Cochrane were searched for relevant studies published from January 1946 to May 2013. Randomised controlled or crossover studies which measured any of these parameters in untrained but otherwise healthy participants in the 48 h following about of exercise, less than 1 h in duration were included.

**Results:**

Ten studies met the inclusion criteria. The results indicate a single bout of aerobic or resistance exercise of moderate to high intensity promotes an increase in IL-6 (145 %) and neutrophil counts (51 %). It appears that 30–60 min of moderate to high intensity exercise is necessary to elicit such changes although variables such as the mode, intensity and pattern of exercise also affect the response. The acute response of CRP within the included studies is equivocal.

**Conclusions:**

Although responses to CRP are inconsistent, a single bout of exercise can increase the activity of both circulating IL-6 and neutrophil counts in untrained adults. These immune and inflammatory responses to a single bout of exercise may be linked to a range of health benefits.

## Key Points

- IL-6 and neutrophil counts increase in response to a bout of aerobic or resistance exercise of moderate to high intensity lasting 30–60 min in duration.
- The acute effect of a single bout of exercise on C-reactive protein is equivocal, and further research is warranted.
- Transient changes in immune and inflammatory markers evoked by a single bout may be linked to the health benefits of regular exercise.

## Background 

Physical inactivity is an established independent risk factor for cardiovascular disease (CVD) [36, 71]. CVD is the major cause of mortality within developed nations and at the forefront of this disease pathology is chronic systemic low-grade inflammation [29]. Characteristically, this state of inflammation permits the secretion of pro-inflammatory cytokines particularly IL-6, tumour necrosis factor (TNF)-α and CRP which are actively involved in insulin resistance and hyperglycaemia [74]. A likely downstream effect of hyperglycaemia is endothelial dysfunction which is associated with a reduction in the bioavailability of vasodilators such as nitric oxide (NO) [1, 13] and is often regarded as an initial step in the development of CVD [62]. Such detrimental effects inhibit the key functions of endothelial cells, namely modulating vascular tone, and over time may lead to cardiovascular complications [6].

Achieving current physical activity recommendations which encourages daily exercise of at least moderate intensity and accumulating 150 min per week may be an effective strategy to reduce the risk of CVD and other diseases [18]. Regular physical activity promotes many health benefits including improved glucose disposal, reduced blood pressure and favourable changes in the blood lipid profile, all of which have beneficial effects on CVD risk [14, 46]. At any stage, individuals adhering to the physical activity guidelines are likely to be no more than 48 h from their last bout of physical activity. Therefore, the beneficial adaptations derived from physical activity may be in part, attributed to the short-term or acute changes that occur in the minutes, hours and days following a bout of activity [27]. Thus, a single bout of exercise appears to act as a stimulus for changes which cumulatively are regarded as exercise adaptations.

Physical activity involves the contraction of skeletal muscles [25] which may promote the synthesis and secretion of anti-inflammatory cytokines and peptides from myotubes commonly termed as ‘myokines’ [56]. Research to date indicates that a single bout of moderate to vigorous intensity aerobic exercise lasting 30–60 min in duration stimulates muscle-derived IL-6 [26, 35, 48, 49, 65, 68]. However, findings in this area are equivocal with others reporting no transient changes following a bout of exercise [19, 21, 43, 47]. The differing outcomes may be attributed to methodological issues, such as exercise intensity or duration or indeed the individual characteristics of the sample population particularly training status.

Elevated plasma concentrations of muscle-derived IL-6 subsequently initiates the secretion of IL-1 receptor agonist and IL-10 from monocytes and lymphocytes [69]. An increase in the appearance of such anti-inflammatory cytokines promotes a range of benefits in vascular reactivity, lipid and glucose metabolism and the suppression of pro-inflammatory cytokines which may reduce the incidence of disease [55]. Exercise-induced changes in IL-6 may also promote an increase in CRP within 24 h of exercise cessation [57]. Although CRP is largely pro-inflammatory, the immediate post-exercise anti-inflammatory actions may also promote endothelial homeostasis by inhibiting cytokines involved in leukocyte activation, proliferation and endothelial dysfunction [57].

The chronic adaptations gained from exercise training are reasonably well established, however, the acute effects have received less attention. Much of the acute effect research to date has focused largely upon either trained or clinical populations, unconventional bouts of activity, testing in the postprandial state or the addition of co-interventions such as antioxidant supplementation. Therefore, no research to date exists regarding the acute response to a bout of exercise within an untrained population or any discussion of the potential mechanisms that confer anti-inflammatory effects, highlighting a clear gap for this review. Moreover, previous work has arguably focused on studies with modes, intensities and durations of exercise that are largely unachievable and unappealing for members of the general public [52, 54, 70].

Given that a large proportion of the population could arguably fall into the category of untrained [77], the findings of this review may help to inform future physical activity recommendations to combat the burden of hypokinetic conditions. Delineating this response would also assist in identifying optimal volumes of exercise required to activate anti-inflammatory mechanisms and a range of health benefits that may prevent disease. The purpose of this review was to examine and synthesise the acute effects of exercise with reference to current guidelines on selected immune and inflammatory markers in untrained adults. In addition, this review will proceed to explore the possible novel physiological mechanisms, through which exercise may confer any observed changes.

## Methods

A computerised systematic search was conducted from January 1946 to May 2013 on the following databases: MEDLINE, EMBASE, CINAHL, Sports Discus and Cochrane. The search terms used include ‘exercise’, ‘acute exercise’, ‘IL-6’, ‘C-reactive protein’, ‘neutrophil activation’, ‘neutrophilia’ or ‘leukocytosis’. Searching was limited to adult human trials and those in English language. Studies that met the following criteria were included: (i) healthy, untrained and fasted (at least 8 h) participants; (ii) a bout of aerobic, anaerobic or resistance exercise less than or equal to 1 h in duration; (iii) at least one outcome measured in blood prior to and following exercise and (iv) randomised resting controlled or crossover trials. Hand searching of original articles was also performed. Studies analysing the effects of exercise in fasted participants were selected as the postprandial state is a confounding variable known to influence markers of inflammation [39, 51]. Articles were excluded if they reported eccentric exercise or any co-intervention. In addition, studies that classified participants as trained (greater than 2 h moderate intensity exercise per week or a mean age related VO_2_max greater than excellent as classified by the ACSM [3]) were excluded. The following data was extracted: participant numbers, age, gender, body mass index (BMI), training status, VO_2_max, exercise trial details and pre- and post-exercise values for the variables of interest (IL-6, CRP and neutrophil counts). Acute messenger ribonucleic acid (mRNA) responses to exercise were deemed to be outside the scope of this review.

### Quality Assessment

The methodological quality of each study was assessed using the Cochrane Collaboration Handbook (2011). After consulting the specified criteria, a ‘low’, ‘uncertain’ or ‘high’ risk of bias was subjectively assigned to each study for the following: selection, performance, detection, attrition, reporting and other bias. The allocation of risk was affected by the quality of reporting as outlined by Halbert and colleagues [24]. Two authors (WB and MM) assessed the risk of bias independently with any disagreements resolved by consensus.

## Results

### Studies Selected

**Fig. 1:**
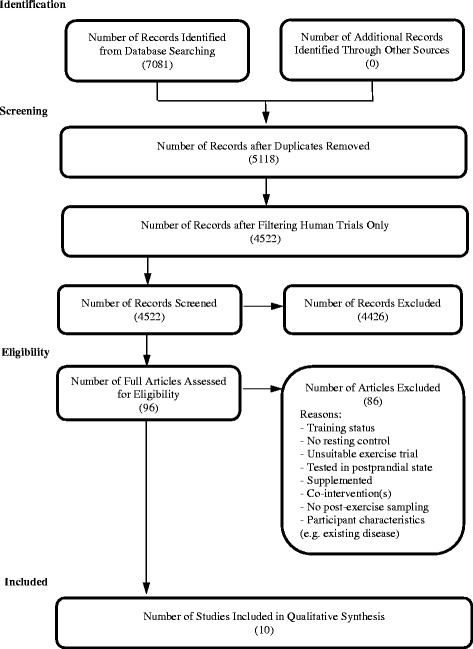
PRISMA schematic summarising the process of data collection

**Fig. 2:**
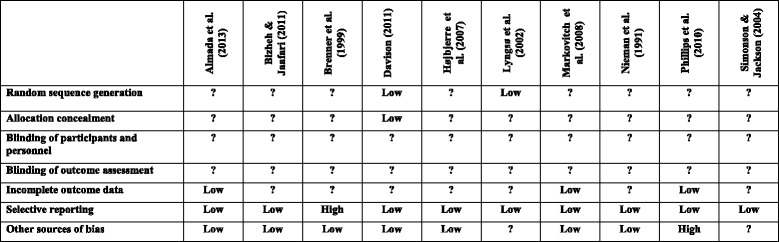
Risk of bias summary for included studies

A PRISMA schematic for the searching strategy is outlined in Fig. 1. Initial searches revealed 96 original articles as possible studies for inclusion. Ten studies met the inclusion criteria and were eligible for inclusion in the current review [2, 4, 7, 16, 28, 40, 43, 50, 58, 66]. A summary of each study, including baseline to post-exercise responses, can be observed in Table 1. All studies incorporated a resting control group alongside one or two suitable exercise trials of differing intensity, pattern and/or duration. Two studies had a ‘high’ risk of bias rating for reporting and other biases, although all remaining studies were categorised as having an ‘unclear’ or ‘low’ risk of bias (see Fig. 2).

**Table 1:** Summary of acute effects of exercise on inflammatory markers in healthy untrained individuals

Name (year)	Design	Subjects			Details of exercise trial	Sample times	Outcome	Reported change
		Group	*n* (gender)	Age (y)				
Nieman et al. (1991) [50]	RCOT	Exercise	12 (F)	36.9 ± 2.2	Walking at 60 % VO_2_max for 45 mins	Pre, P 0 and 1.5 h	NC	Increased from pre to P 0 h between groups
		Control						
Brenner et al. (1999) [7]	RCOT	Exercise	8 (M)	24.9 ± 2.3	Cycling at 90 % VO_2_max for 5 mins	Pre, P 0, 3, 24 and 72 h	IL-6	No change within or between conditions
		Exercise			Circuit (5 exercises; 3 sets × 10 reps) at 60–70 % 1RM			
		Control						
Lyngsø et al. (2002) [40]	RCT	Exercise	9 (8 M)	23.6 ± 0.4	Cycling at 60 % VO_2_max for 1 h	Pre, P 0 and every 30 mins to 3 h	IL-6	Increased from Pre at P 0, 0.5, 1.5 and 2 h within the exercise condition
		Control	7 (5 M)	24.0 ± 0.3				
Simonson and Jackson (2004) [66]	RCT	Exercise	8 (M)	30 ± 7	Resistance (8 exercises; 3 sets × 8–10 reps) at 75 % 1RM for 45 mins	Pre, P 0, 15 and 30 mins	NC	Increased from Pre to P 0, 15 and 30 mins within and between groups
		Control	8 (M)					
Højbjerre et al. (2007) [28]	RCOT	Exercise	16 (M)	26.3 ± 0.8	Cycling at 55 % VO_2_max for 1 h	Pre, P 0 and every 30 mins to 2.5 h	IL-6	Increased from Pre to P 0 and 2.5 h within the exercise group
		Control		26.0 ± 0.7				
Markovitch et al. (2008) [43]	RCOT	Exercise	12 (M)	54 ± 4	Walking at 50 % VO_2_max for 30 mins	Pre, P 0, 2, 24, 48, 72 and 168 h	IL-6, CRP and NC	No change within or between conditions
		Control						
Phillips et al. (2010) [58]	RCOT	Exercise 1	14 (M)	21.7 ± 1.7	Resistance exercise (2 sets of 12 reps at 65 % 1RM then 3rd set to fatigue)	Pre and P 0 h	IL-6	Increased from Pre to P 0 h with both intensities compared to control
		Exercise 2			Resistance exercise (2 sets of 8 reps at 85 % 1RM then 3rd set to fatigue)			
		Control						
Bizheh and Jaafari (2011) [4]	RCT	Exercise	14 (M)	44.9 ± 4.1	Circuit (10 exercises × 3 sets) at 35 % 1RM totaling 10 mins	Pre and P 0 h	CRP	Increased from Pre to P 0 h within the exercise group
		Control	9 (M)	43.1 ± 5.2				
Davison (2011) [16]	RCOT	Exercise	9(M)	27 ± 5	Cycling Wingate Test (4 × 30 s)	Pre, P 0 and 30 mins	NC	Increased from Pre to P 0 and 30 mins within and between groups
		Control						
Almada et al. (2013) [2]	RCT	Exercise	5 (M)	20.6 ± 2.1	Running submaximally for 45 mins followed by 7 mins at 90 % VO_2_max	Pre and P 0 h	IL-6	Increased from Pre to P 0 h within and between groups
		Control	5 (M)					

*RCOT* randomised crossover trial, *RCT* randomised control trial, *n* number of participants, *F* female, *M* males, *y* years of age, *mins* minutes, *1 RM* 1 repetition maximum, *h* hour(s), *Pre* pre-exercise, *P* post-exercise, *NC* neutrophil count, *IL-6* interleukin-6, *CRP* C-reactive protein

### Participant Characteristics

Sample populations in the studies included varied from 8 to 23 with an average of 14 participants. Overall, 136 healthy participants were evaluated with 107 undertaking experimental exercise trials and 100 acting as control participants (73 of which acted as their own control). Eight studies examined exclusively male participants, one exclusively female, whilst the remaining study assessed a mixed gender sample. In total, 121 men and 15 women participated in the studies. The mean age of all participants was 31 ± 11 years (range, 21–54 years) whilst mean BMI was 25 ± 2 kg/m^2^ (range, 23–28 kg/m^2^). Some studies did not directly record individual BMI so this was estimated using reported mean anthropometric measurements. The training status of the participants ranged from sedentary (*n* = 1) or inactive (*n* = 2) to recreationally active (*n* = 5). The training status was not specifically mentioned in two studies but analysis of VO_2_max data suggests that the participants were active but not trained. Lastly, in those studies that measured maximal oxygen consumption (*n* = 7), the mean VO_2_max was 37.5 ± 10.3 ml·kg^−1^·min^−1^ (range, 22–49 ml·kg^−1^·min^−1^) indicating that all the participants were untrained.

### Characteristics of Exercise Trials

Twelve exercise trials met the inclusion criteria. Four studies examined the impact of aerobic exercise as the sole experimental trial utilising walking (*n* = 2) and cycling (*n* = 2) as the exercise mode with all reporting exercise intensity as a percentage of VO_2_max. The mean duration and intensity of the walking trials was 37.5 min (range, 30–45 min) and 55 % VO_2_max (range, 50–60 % VO_2_max), respectively. The mean duration and intensity of the cycling trials was 1 h and 57.5 % VO_2_max (range, 55–60 % VO_2_max), respectively. Two studies analysed the effects of anaerobic cycling exercise. The mean duration of exercise was 2.5 min (range, 2–5 min), and the exercise intensity prescribed was equal to or greater than 90 % VO_2_max. Five exercise protocols assessed the effects of resistance exercise that focused on all major muscle groups (extremities, core, chest and back) with rest periods between sets. The mean duration of the resistance trials was 28.5 min (range, 12–45 min; *n* = 2 [no duration specified in two studies]) with all reporting an exercise intensity related to a percentage of 1 repetition maximum (1RM). Therefore, the mean exercise intensity for resistance trials was 65 % 1RM (range, 35–85 % 1RM). The remaining study analysed the effects of a combined running exercise trial. The total exercise duration was 52 min (45-min aerobic exercise followed by 7-min anaerobic exercise), and the exercise intensity was set at 60 % VO_2_max (aerobic) and 90 % VO_2_max (anaerobic), respectively.

### Acute IL-6 Response

Six studies, consisting of eight exercise trials, measured IL-6 in plasma or serum at baseline and either immediately post-exercise or within 5 min. Two studies [7, 43] reported no change within or between trials. Conversely, three studies [2, 28, 40] reported a significant increase within the exercise condition from baseline to post-exercise. IL-6 concentrations remained significantly elevated over time within two of these studies as Lyngsø et al. [40] and Højbjerre et al. [28] reported increased concentrations at 2 and 2.5 h respectively. Two further studies [2, 58] reported significant differences between the control and exercise groups post-exercise whereas another study reported no significant differences between conditions [25]. Within those studies that reported changes, the relative increase in IL-6 between baseline and post-exercise was 145 %.

### Acute CRP Response

Two studies [4, 43] measured serum CRP at baseline and post-exercise. One study [4] reported no difference between exercise and control conditions but observed a significant difference within the exercise group from baseline to post-exercise. The other study [43] reported no significant differences within or between conditions over time.

### Acute Neutrophil Response

Four studies [16, 43, 50, 66] measured changes in neutrophil counts prior to and following exercise. One study [43] reported no significant difference within or between conditions from baseline to post-exercise and over time. The remaining three studies reported significant differences from baseline to post-exercise within the exercise trial [16, 66] and between [16, 50, 66] exercise and control conditions. All three studies also reported significant differences over time between conditions at 30 min [16, 66] and 3 h [50] post-exercise. The relative increase in neutrophil count from baseline to post-exercise was 51 %.

## Discussion

This systematic review aimed to consider acute changes in certain immune and inflammatory markers following a bout of exercise in untrained participants. The results suggest that IL-6 and neutrophil counts increase in response to exercise. The post-exercise response of CRP is unclear due to the lack of studies and conflicting findings making firm conclusions difficult to ascertain.

### Acute IL-6 Response

IL-6 was measured in six of the studies reviewed. Four studies, consisting of five exercise trials, reported a significant change in IL-6 from baseline to post-exercise. Almada et al. [2] reported that IL-6 increased from 1.1 ± 0.6 pg/ml at baseline to 2.7 ± 1.0 pg/ml immediately post-exercise whilst there was no change within the control group. In agreement, both Højbjerre et al. [28] and Lyngsø et al. [40] reported a similar change in IL-6 which remained elevated for at least 2 h post-exercise. The remaining crossover study reported the greatest increase in IL-6. Baseline concentrations for both the lower and higher intensity exercise bouts were similar at 2.5 ± 0.4 and 2.8 ± 0.4 pg/ml, respectively, with post-exercise concentrations of 7.4 ± 1.3 and 5.2 ± 0.7 pg/ml [58].

The mechanisms behind muscular IL-6 synthesis and secretion remain unclear although a plausible explanation may be attributable to cellular stress. Kramer and Goodyear [31] suggest that an exercise-induced increase in reactive oxygen species (ROS) augments IL-6 protein secretion and gene transcription in part due to the activation of mitogen-activated protein kinase (MAPK) and NF-_κ_B signalling within skeletal muscle. NF-_κ_B is a redox-sensitive transcription factor comprised of five proteins (p50, p52, p65, Rel B and c-Rel), and it exists within the cytoplasm as an inactive heterodimer whilst bound to the inhibitory subunit IkB [59]. Increased concentrations of ROS can activate IkB-α kinase (IKK) which results in the phosphorylation and degradation of IkB allowing NF-_κ_B subunits to dimerise, promoting nuclear translocation and possibly cytokine gene expression [60]. ROS have been identified as important modulators of cell signalling and they have been shown to increase following a single bout of aerobic exercise [17] possibly due to mitochondrial electron leakage, enhanced neutrophil recruitment, catecholamine auto-oxidation and the involvement of several enzymes including NADPH oxidase, xanthine oxidase and phospholipase A_2_ [23, 59]. Similarly, it has been shown that NF-_κ_B transiently increases in response to a single bout of intense resistance exercise in humans [76]. Although entirely speculative, it is possible that an accumulation of ROS could activate NF-_κ_B however, more research is required to support this hypothesis. No study within this review directly measured ROS and NF-_κ_B, but it may be possible that the exercise stimulus adopted may have provided ideal conditions for the formation of ROS and downstream increases in IL-6.

The exercise stimulus, particularly exercise duration and intensity has often been cited as key elements in stimulating IL-6 synthesis and secretion by skeletal muscles [53, 57]. Three out of the four studies that reported significant differences in IL-6 employed aerobic exercise protocols lasting approximately 1 h in duration. In addition to the exercise stimulus, the muscle mass recruited and physical fitness have also been postulated to affect plasma IL-6 concentrations [57]. As the participants are untrained, the extended duration and intensity of exercise may promote a reduction or depletion in intramuscular glycogen. Untrained skeletal muscles are less adept at conserving glycogen stores, and as a consequence, this stimulates the upregulation of IL-6 expression [30] possibly in some part due to the enhanced activity of AMP-activated protein kinase (AMPK) [41] or skeletal muscle mRNA expression. Louis et al. [38] and Buford et al. [8] confirmed this by reporting increased IL-6 mRNA expression following a single bout of moderate to vigorous intensity exercise in recreationally active participants. Therefore, enhanced gene expression may result in the observed increase in plasma concentrations of the functional IL-6 protein. The remaining study that reported post-exercise changes assessed the IL-6 response to both high-intensity low-volume and low-intensity high-volume resistance exercise. The results from this study indicate that a greater exercise volume promotes a greater increase in IL-6 concentration although the exercise mode and intensity is also a contributory factor.

Although the precise mechanisms remain unclear, an increase in circulating IL-6 following exercise is an important physiological response, and it has been suggested that IL-6 has many biological functions. A downstream health benefit of a transient increase in muscle-derived IL-6 is an increase in glucose and lipid metabolism [22, 55]. Carey and colleagues [12] reported that infusion of rhIL-6 increased the phosphorylation of AMPK in healthy humans and stimulated glucose disposal and GLUT-4 translocation. In addition, AMPK phosphorylates acetyl-CoA carboxylase (ACC) which in turn decreases malonyl CoA and removes the inhibition on carnitine palmitoyltransferase (CPT)-1 resulting in fatty acid oxidation [72]. Such benefits promote greater metabolic control and may assist in reducing the incidence of metabolic disease over time. Moreover, a transient increase in exercise-induced IL-6 seems to have an inhibitory effect on the activity of pro-inflammatory cytokines particularly TNF-α and IL-1β [57]. IL-6 is believed to stimulate a cascade of anti-inflammatory cytokines and receptors particularly IL-10 alongside TNF-α and IL-1 receptors which subsequently neutralise these potent pro-inflammatory cytokines [57]. This response prevents TNF-α from interfering in insulin signalling and may prevent hyperglycaemia and metabolic disorder [57].

In contrast, two studies reported no significant differences in IL-6 from baseline to post-exercise. These conflicting findings may be attributed to various methodological issues. Both studies recruited relatively small sample sizes (*n* = 8 [7] and 12 [43]) possibly resulting in a lack of statistical power. Additionally, the exercise stimulus adopted may be responsible for the lack of difference within the findings. As previously indicated, IL-6 upregulation is dependent upon exercise intensity and duration, but Markovitch et al. [43] employed 30 min of moderate intensity exercise (50 % VO_2_max) whilst Brenner et al. [7] examined both a moderate intensity circuit trial equivalent to 50 % VO_2_max and short-duration high-intensity cycling (5 min) which may not be of sufficient intensity to evoke an anti-inflammatory response.

### Acute CRP Response

CRP is an independent marker of CVD risk, and it was measured in two of the studies reviewed. Bizheh and Jaafari [4] reported that CRP increased from 1.98 ± 0.46 mg/l at baseline to 2.67 ± 0.5 mg/l post-exercise whilst there was no change within the control group. Conversely, Markovitch et al. [43] reported no change in CRP following exercise. The equivocal findings between the two studies may be attributed to numerous methodological factors though the only apparent differences lie in the exercise stimulus and the percentage body fat of the participants involved.

CRP is an acute phase reactant that tends to only increase (within the exercise model) following a strenuous or prolonged bout of exercise or exercise resulting in muscular injury [5, 64]. This challenges the findings of Bizheh and Jaafari [4] as a low-intensity resistance exercise protocol was employed and supports the results reported by Markovitch et al. [43] as daily accustomed moderate-intensity exercise should not present increases in CRP. In agreement, studies ineligible for inclusion in the current review adopted similar exercise protocols and participants but reported no change in CRP immediately following and up to 24 h post-exercise [15, 32, 44, 47]. A possible explanation for the rise in CRP reported by Bizheh and Jaafari [4] may be that the resistance protocol induced muscular damage as the participants are untrained and may be unaccustomed to this mode of physical exertion. CRP plays a key role in recognising pathogens and cell debris so increased concentrations may be linked to the leakage of muscular components into circulation [5]. An alternative mechanism responsible for exercise-induced increases in CRP may be the parallel increase in anti-inflammatory cytokines. An increase in IL-6 is thought to promote the hepatic synthesis and secretion of CRP [5] although only one of the two studies measured IL-6 and reported no changes so it is difficult to determine the exact mechanism responsible.

Lin and colleagues [37] have demonstrated that an increase in CRP is strongly associated with percentage of body fat mass, and this association is linked to increased adipose tissue. Adipose tissue secretes several adipokines which mediate inflammation and promote the hepatic synthesis of CRP and release into circulation [57]. The mean percentage body fat for participants recruited by Bizheh and Jaafari [4] was 26.3 ± 4.4 % (exercise group) and 28.2 ± 5.4 % (control) whereas the mean for participants recruited by Markovitch et al. [43] was 22 ± 2 %. Consequently, this apparent difference may account for the conflicting concentration of CRP between the two studies following a single bout of exercise.

### Acute Neutrophil Response

Blood neutrophil count was measured in four of the studies reviewed. Nieman et al. [50] reported neutrophil counts increased from 3.99 ± 0.21 × 10^9^ cells·L^−1^ at baseline to 5.36 ± 0.41 × 10^9^ cells·L^−1^ immediately post-exercise. Simonson and Jackson [66] reported a similar increase in neutrophils following resistance exercise which remained elevated for 30 min. In agreement, Davison [16] reported that neutrophil count increased from 2.3 ± 0.7 × 10^9^ cells·L^−1^ at baseline to 4.0 ± 1.5 × 10^9^ cells·L^−1^ following a bout of high intensity exercise which also remained elevated for 30 min. This response appears to be a common finding as ineligible studies for the current review have also reported increased neutrophil counts following aerobic and maximal bouts of exercise in similarly untrained participants [42, 61]. The increase in blood neutrophil counts appears to be less sensitive to the exercise stimulus as a similar response is evident across a range of intensities, durations and exercise modes.

Neutrophils are the primary leukocyte subpopulation to arrive at damaged or stressed tissue sites [9]. Previously, it was thought that the increase in the number of neutrophil cells is directly linked to exercise-induced muscular damage although similar observations have been reported following non-damaging exercise [68]. Therefore, the likely mechanisms responsible for this increase are multifactorial and may include an increase in the release of various biochemical products such as catecholamine’s or alternatively enhanced cell signalling or blood flow [9].

Skeletal muscles can recruit neutrophils via cytokine signalling, but once they penetrate the cell membrane, neutrophils engage in phagocytosis commonly referred to as the ‘respiratory burst’. Activated neutrophils produce a range of ROS including superoxide (O_2_^•−^) and undergo a process of degranulation [23]. Antioxidant enzymes reduce the potency of O_2_^•−^ by dismutating it to hydrogen peroxide, (H_2_O_2_) but in the presence of myeloperoxidase, a degranulation enzyme, another potent oxidant is produced in the form of hypochlorous acid (HOCl) [63]. This suggests that increased cell counts of neutrophils may be undesirable for cardiovascular health as they have been implicated in cellular damage and endothelial dysfunction via an oxidative stress mechanism. Alternatively, increased neutrophil cell recruitment following exercise may also have beneficial effects upon health, as they are involved in muscular regeneration possibly via the activation of satellite cells and their recruitment usually activates other immune cell subpopulations such as lymphocytes [49]. An increase in the recruitment of such cells following exercise promotes the upregulation of anti-inflammatory gene expression and proteins, particularly IL-1 receptors [10] which suppress the pro-inflammatory cytokine IL-1β. In addition, H_2_O_2_ is now recognised as an important signalling molecule if it can avoid decomposition by antioxidants [20, 75] and it may be important for exercise-derived health benefits particularly those relating to the modulation of endothelial cell function [11].

In contrast, Markovitch et al. [43] reported no change in neutrophils following moderate intensity aerobic exercise. This may be attributed to the exercise stimulus however, an ineligible study by Nieman and colleagues [49] adopted a similar experimental trial and reported a significant increase in neutrophil counts immediately post-exercise. Although the differing number of participants or alternatively the slightly greater exercise volume employed (60 % VO_2_max) may account for this discrepancy in neutrophil cell recruitment.

### Limitations

The systematic review presented is not without limitations. Studies favoured male participants as the sample population and as such, the results are more generalised to this specific gender. The choice to select male participants may be influenced by the biochemical effects of the menstrual cycle [67], and researchers may have wished to avoid these complications by selecting a convenience sample. Nevertheless, it is not implausible to suggest that similar immune and inflammatory health benefits might accrue for females undertaking similar bouts of exercise.

A further limitation was the concept of ‘untrained’ participants, as this was difficult to define. Some studies used terminology such as ‘sedentary’ or ‘inactive’ to describe the participant training status whereas others used ‘physically’ or ‘recreationally active’. The inclusion criteria for training status was set at less than or equal to 2 h as less than half the UK adult population achieve recommended activity levels [73]. This postulates that the results are more representative of typical physical activity patterns. It was important to distinguish the training status as previous reports have shown an inverse relationship between physical fitness and certain markers of inflammation [33, 34].

Finally, the studies included adopted differing exercise protocols in terms of mode, duration and intensity as well as the timing of post-exercise sampling. This means that it is difficult to draw consistent conclusions with regard to the exact physical activity recommendations necessary to elicit the health benefits discussed. Furthermore, the acute health benefits associated with a single bout of exercise and the proposed physiological mechanisms explored within this review are speculative and further research is warranted.

## Recommendations for Future Research

Despite an increase in the number of studies looking at the association between exercise and health, the transient health benefits of exercise remain poorly understood. Future studies should address the paucity of research examining female participants as well as the lack of randomised control or crossover trials incorporating a ‘true’ resting control. Tackling both these issues will provide an insight into the beneficial or detrimental effects of a single bout of exercise on CVD risk factors in females and whether any health benefits conferred are solely attributed to exercise. In order to resolve these issues, researchers should carefully devise and implement robust study designs accounting for the main confounding variables, which may serve to elucidate the specific contribution of individual bouts of exercise to overall health.

In addition, the exercise characteristics or stimuli should gain more focus, as this inevitably provides the stress that promotes the acute responses. Future research should explore the effects of differing exercise intensities and durations possibly across different populations and how these relate to transient changes in immune cells, cytokine concentrations and endothelial function. Equally, the mode of exercise warrants further investigation to clarify any differences between aerobic and resistance methods and which promotes the greatest benefits. Moreover, it may also be of interest to evaluate the impact of exercise trials tailored for members of the general public. Walking has previously been described as the cornerstone of physical activity interventions and yet the acute health benefits gained from this type of activity are not fully understood [45]. Establishing optimal doses of exercise that augment a range of health benefits may inform physical activity prescription guidelines and possibly reduce the incidence of disease.

Additionally, further research is required to elucidate the time course of acute exercise-induced responses. In order to accomplish this, researchers should incorporate the use of multiple post-exercise measurements within the study design. Not only will this allow the tracking of markers but may also contribute to the identification of optimal measurement times. Finally, future investigations should also attempt to clarify the physiological mechanisms whereby acute responses in skeletal muscle-derived cytokines relate to exercise training adaptations and health in general. A potential strategy for accomplishing this may be to assess multiple accepted markers of cardiovascular health particularly those involved in cell signalling, metabolism and gene expression.

## Conclusions

A single bout of either aerobic or resistance exercise with a moderate to vigorous/high intensity promotes an increase in systemic concentrations of muscle-derived IL-6 and circulating neutrophil counts. The health benefits achieved from this IL-6 response assist in the clearance of glucose and lipoproteins from circulation and improve insulin sensitivity and may prevent the initiation and development of CVD as both macronutrients are integral in the development of the fatty streak and atherosclerotic plaque. Accordingly, physical activity recommendations may encourage untrained adults to undertake bouts of moderate to vigorous aerobic or resistance exercise greater than 30 min in duration. More parallel research is required to establish the effects on CRP as there is a lack of evidence and conflicting findings.
